# New resilience instrument for family caregivers in cancer: a multidimensional item response theory analysis

**DOI:** 10.1186/s12955-021-01893-8

**Published:** 2021-11-18

**Authors:** Mu Zi Liang, Ying Tang, Peng Chen, Jian Liang, Zhe Sun, Guang Yun Hu, Yuan Liang Yu, Zeng Jie Ye

**Affiliations:** 1 Guangdong Academy of Population Development, Guangzhou, 510600 Guangdong Province China; 2 Institute of Tumor, Guangzhou University of Chinese Medicine, Guangzhou, 510006 China; 3 Basic Medical School, Guizhou University of Traditional Chinese Medicine, Guiyang, 550025 China; 4 Guangdong Provincial Key Laboratory of New Drug Development and Research of Chinese Medicine, Mathematical Engineering Academy of Chinese Medicine, Guangzhou University of Chinese Medicine, Guangzhou, 510006 China; 5 The First Affiliated Hospital, Guangzhou University of Chinese Medicine, Guangzhou, 510405 Guangdong Province China; 6 Army Medical University, Chongqing Municipality, 400038 China; 7 South China University of Technology, Guangzhou, 510641 Guangdong Province China; 8 Guangzhou University of Chinese Medicine, Guangzhou, 510006 Guangdong Province China

**Keywords:** Resilience, Instrument, Family caregivers, Cancer, Multidimensional item response theory, Generalized additive model, Latent profile analysis

## Abstract

**Objective:**

Resilience instruments specific to family caregivers (FCs) in cancer are limited. This study was designed to validate the 10-item Resilience Scale Specific to Cancer (RS-SC-10) in FCs using multidimensional item response theory (MIRT) analysis.

**Methods:**

382 FCs were enrolled from Be Resilient to Cancer Program (BRCP) and administered with RS-SC-10 and 36-item Short Form Health Survey (SF-36). MIRT was performed to evaluate item parameters while Generalized Additive Model (GAM) and Latent Profile Analysis (LPA) were performed to test the non-linear relationship between resilience (RS-SC-10) and Quality of Life (QoL, SF-36).

**Results:**

RS-SC-10 retained 10 items with high multidimensional discrimination, monotonous thresholds and its original two-factor structure (*Generic* and *Shift-Persist*). Four latent resilience subgroups were identified and a non-linear dose–response pattern between resilience and QoL was confirmed (per-SD increase OR = 1.62, 95% CI 1.16–2.13, p = 0.0019).

**Conclusion:**

RS-SC-10 is a brief and suitable resilience instrument for FCs in cancer. The resilience screening of patients and FCs can be performed simultaneously in clinical practice.

**Supplementary Information:**

The online version contains supplementary material available at 10.1186/s12955-021-01893-8.

## Introduction

Advances in new therapies of cancer (i.e., immunotherapy) have resulted in significant improvements in survival rates, and cancer is gradually treated as a chronic disease [1, 2]. However, cancer survivors are still troubled with physical (i.e., fatigue, etc.), psychosocial (i.e., fear of cancer recurrence, etc.), and financial burdens(i.e., in debt, etc.) in the remission stage and family caregivers (FCs) are considered to play an essential role in the cancer survivorship [3, 4]. Although FCs are valuable sources of support to cancer survivors, they also have heavy caregiver burdens about monitoring treatment sessions, managing symptoms and providing emotional support [5]. Therefore, FCs are vulnerable to role strain and stress resulting in high risks for morbidity and mortality [6]. However, a significant group of FCs have the ability to ‘bounce back’from adversity after a short period of disruption, and find meaning and benefits in the role of caregiver. This ability is defined as resilience and FCs with high resilience levels were reported to experience low emotional distress and caregiver burden, as well as high optimism and Quality of Life (QoL) [7]. However, there exist no resilience scales specific to FCs in cancer, and whether a generic resilience instrument can be administered in the cancer-specific population is debated [8]. Recently, we developed a new Resilience Scale Specific to Cancer (RS-SC) based on Shift-Persist theory and Resilience Model to Breast Cancer [9, 10]. Then, a 10-item version (RS-SC-10) with high item functions was developed based on Item Response Theory (IRT) analysis [11, 12], and validated in our Be Resilient to Breast Cancer (BRBC) program [13, 14]. Thus, we have interests whether this powerful resilience instrument can also be applied to FCs, which will provide important information for resilience screening in clinical practice. Furthermore, RS-SC-10 could also be used as a composite index to evaluate FCs' psychosocial functions and assess the efficacy of resilience-related intervention in future studies. In the current study, a Multidimensional Item Response Theory analysis (MIRT, also called as full information analysis) was performed to evaluate the factor structure and item functions of RS-SC-10 with a sample of FCs in cancer [15]. In addition, Generalized Additive Model (GAM) and Latent Profile Analysis (LPA) were performed to test the non-linear relationship between resilience (RS-SC-10) and QoL [16]. In the present study, we hypothesized: (1) RS-SC-10 would retain its original two-factor structure; (2) multidimensional difficulty of RS-SC-10 would be distributed monotonously; (3) 10 items of RS-SC-10 would show high multidimensional discriminative abilities against caregivers with different resilience levels; (4) several distinct resilience patterns would be identified by LPA; (5) there existed a non-linear dose–response relationship between resilience and QoL.

## Methods

### Participants and data collection

Participants were recruited from our Be Resilient to Cancer Program (BRCP) between July 2016 and November 2017 and was approved by the Human Research Ethics Committee (No.2016KYTD08) [13, 14, 17, 18]. The inclusion criteria were: (1) Family caregivers (FCs), (2) their relatives had a confirmed diagnosis of cancer, (3) aged > 18 years, (4) could communicate in Mandarin or Cantonese fluently. The exclusion criteria were: (1) linguistic or intellectual difficulties, (2) had a currently active Axis I psychiatric disorder, (3) unwilling to participate in the study. Three full time research nurses were trained to approach potential FCs and a standardized face-to-face interview was performed to collect baseline information as well as informed consent.

### Sample size

A consensus has not been reached about the optimal sample size for MIRT analysis. In the current study, the sample size is based on Linacre's recommendations that a sample size of n = 300 will be a robust estimation of item parameters (within 0.5 logits [contraction of log-odds probability units] at α = 0.01) with a minimum dropout of 15% on the basis of data from previous research [19]. Thus, 382 was efficiently powerful to perform the MIRT analysis.

### Instruments

#### 10-item Resilience Scale Specific to Cancer (RS-SC-10)

The original RS-SC is a 25-item resilience instrument specific to cancer that has the five domains of generic element, benefit finding, support and coping, hope for the future, and meaning for existence [9]. A 10-item RS-SC (RS-SC-10) was later developed based on MIRT analysis and two dimensions were retained including Generic and Shift-Persist, with higher scores indicating higher resilience levels (score ranges from 10 to 50) [12]. The Cronbach’s α of RS-SC-10 is 0.86. The Minimum Clinical Important Difference for RS-SC-10 is 2 points [20]. RS-SC-25 and RS-SC-10 were attached in the Additional file 1: Table S1 and Additional file 2: Table S2.

#### 36-item Short Form Health Survey (SF-36)

SF-36 is a quality of life (QoL) instrument generic to normal populations [21]. It consists of eight domains that evaluate physical function (PF), general health (GH), role physical (RP), bodily pain (BP), social function (SF), vitality (VT), role emotional (RE) and mental health (MH).The raw score of each dimension was converted to a score ranging from 0 to 100 according to the manual, with higher scores indicating better functional ability. The Cronbach’s α of SF is 0.87. In addition, the value derived from normal populations was utilized as the cut-off for caregivers (coded as 0 for low QoL and 1 for high QoL, respectively) [21].

### Statistical analysis

First, the demographic characteristics of caregivers were presented with descriptive statistics approach. Then, the local independence hypothesis was examined and item-pair local independence was evaluated by residuals correlations (heat maps). A value lower than 0.20 indicated a low risk of systematic fitting problems [22].

Second, based on the two-factor structure of RS-SC in our previous research [12], two models were explored in the current study, including Confirmatory Factor Analysis-based and Bifactor-based MIRT models [15]. A compensatory logistic multidimensional grade response model (MGRM-C) was chosen to estimate the item parameters by the Markov chain Monte Carlo (MCMC) method with a maximum of 4000 cycles, which had been described in details somewhere [12]. MGRM-C is detailed as the equation below:

$$P_{ijk} = \frac{{\exp \left( {{\varvec{a}}_{{\varvec{i}}} {\varvec{\theta}}_{{\varvec{j}}} + d_{ik} } \right)}}{{1 + \exp \left( {{\varvec{a}}_{{\varvec{i}}} {\varvec{\theta}}_{{\varvec{j}}} + d_{ik} } \right)}}$$

MGRM-C is a logistic probability model (*P*_*ijk*_) that examinee (*j*) will respond with category *k* (and above) of item *i* as a function of the item-category threshold (or easiness parameter, *d*_*ik*_), item discrimination parameter vector (*a*_*i*_), and examinee ability parameter vector (*θ*_*j*_). Log-likelihood (LL), Akaike Information Criterion (AIC), Bayesian Information Criterion (BIC) and Sample-adjusted BIC (SABIC) were examined to choose the optimal model. Multidimensional Discrimination (MDISC < 0.5 indicates poor; 0.5–1.0, moderate; 1.0–1.5, good; > 1.5, excellent)) and Multidimensional Difficulty (MDIFF) were calculated as primary indicators to show the multidimensional item capability in distinguishing between individuals with different resilience levels [23]. MDISC > 1.5 and monotonously distributed MDIFF were good fitting indicator to MIRT model. In addition, item trace and item information surface were also visualized to provide additional psychometric characteristics for RS-SC-10 [23]. At last, an iterative hybrid ordinal logistic regression/item response theory approach with Monte Carlo Simulation was performed to estimate Differential Item Functions (DIF) in gender [24].

Third, Latent Profile Analysis (LPA) was utilized to divide resilience-based caregivers into several subgroups and Generalized Additive Model (GAM) was performed to evaluate the non-linear associations between resilience (RS-SC-10) and QoL (SF-36) [16]. Based on LPA-based models, multivariate logistic regressions were used to assess the dose–response patterns between resilience and QoL after controlling the confounders. Crude, adjusted and per-SD OR including 95% CI were evaluated. All statistical methods were run by R and Mplus software. Significance level was 0.05 for all statistical tests.

## Results

### Demographic information

438 caregivers were approached and 56 were excluded for different reasons [“unwilling to participate”(N = 35),“no interests”(N = 15) and “busy schedule” (N = 6)]. No significant difference in gender was identified between the included and excluded caregivers. Among the remaining 382 caregivers, their relatives with lung, gastric, colorectal, and breast cancer diagnoses accounted for 21.2%, 28.3%, 24.1%, and 26.4%, respectively. The majority of caregivers were 40–60 years old (71.5%), spouse (75.4%), unemployment status (61.8%) and had caregiver experience less than 12 months (62.1%). Other details could be found in Table 1.

**Table 1:** Demographic characteristics of informal caregivers categorized by patients’ cancer types (N = 382)

Characteristics (%)	Lung cancer	Gastric cancer	Colorectal cancer	Breast cancer	Total
No	81 (21.2)	108 (28.3)	92 (24.1)	101 (26.4)	382 (100.0)
Sex					
Female	65 (80.2)	65 (60.2)	62 (67.4)	37 (36.6)	229 (59.9)
Man	16 (19.8)	43 (39.8)	30 (32.6)	64 (63.4)	153 (40.1)
Age (years)					
< 40	7 (8.6)	10 (9.3)	4 (4.3)	11 (10.8)	32 (8.3)
40–50	35 (43.2)	61 (56.5)	20 (21.7)	31 (30.7)	147 (38.5)
50–60	25 (30.9)	25 (23.1)	41 (44.6)	35 (34.7)	126 (33.0)
> 60	14 (17.3)	12 (11.1)	27 (29.3)	24 (23.8)	77 (20.2)
Education level					
Middle school or lower	47 (58.0)	69 (63.9)	63 (68.5)	48 (47.5)	227 (59.4)
High school or higher	34 (42.0)	39 (36.1)	29 (31.5)	53 (52.5)	155 (40.6)
Income (CNY/month)					
< 5000	22 (27.2)	33 (30.6)	37 (40.2)	38 (37.6)	130 (34.0)
5000–10,000	42 (51.9)	48 (44.4)	27 (29.4)	42 (41.6)	159 (41.6)
> 10,000	17 (20.9)	27 (25.0)	28 (30.4)	21 (20.8)	93 (24.4)
Relationship to patient					
Spouse	61 (75.3)	87 (80.6)	79 (85.9)	61 (60.4)	288 (75.4)
Non-spouse	20 (24.7)	21 (19.4)	13 (14.1)	40 (39.6)	94 (24.6)
Religious beliefs					
Yes	25 (30.9)	40 (37.0)	22 (23.9)	24 (23.8)	111 (29.1)
None	56 (69.1)	68 (63.0)	70 (76.1)	77 (76.2)	271 (70.9)
Employment status					
Employment	29 (35.8)	34 (31.5)	24 (26.1)	59 (58.4)	146 (38.2)
Unemployment	52 (64.2)	74 (68.5)	68 (73.9)	42 (41.6)	236 (61.8)
Months of caregiving					
< 6	40 (49.4)	19 (17.7)	16 (17.4)	33 (32.7)	108 (28.3)
6–12	24 (29.6)	32 (29.6)	32 (34.8)	41 (40.6)	129 (33.8)
13–24	12 (14.8)	32 (29.6)	27 (29.3)	16 (15.8)	87 (22.8)
> 24	5 (6.2)	25 (23.1)	17 (18.5)	11 (10.9)	58 (15.1)
Combordities					
None	47 (58.0)	49 (45.4)	32 (34.8)	56 (55.4)	184 (48.2)
One	26 (32.1)	45 (41.7)	36 (39.1)	25 (24.8)	132 (34.6)
Two or more	8 (9.9)	14 (12.8)	24 (26.1)	20 (19.8)	66 (17.3)

### Item distribution and local independence

**Fig. 1:**
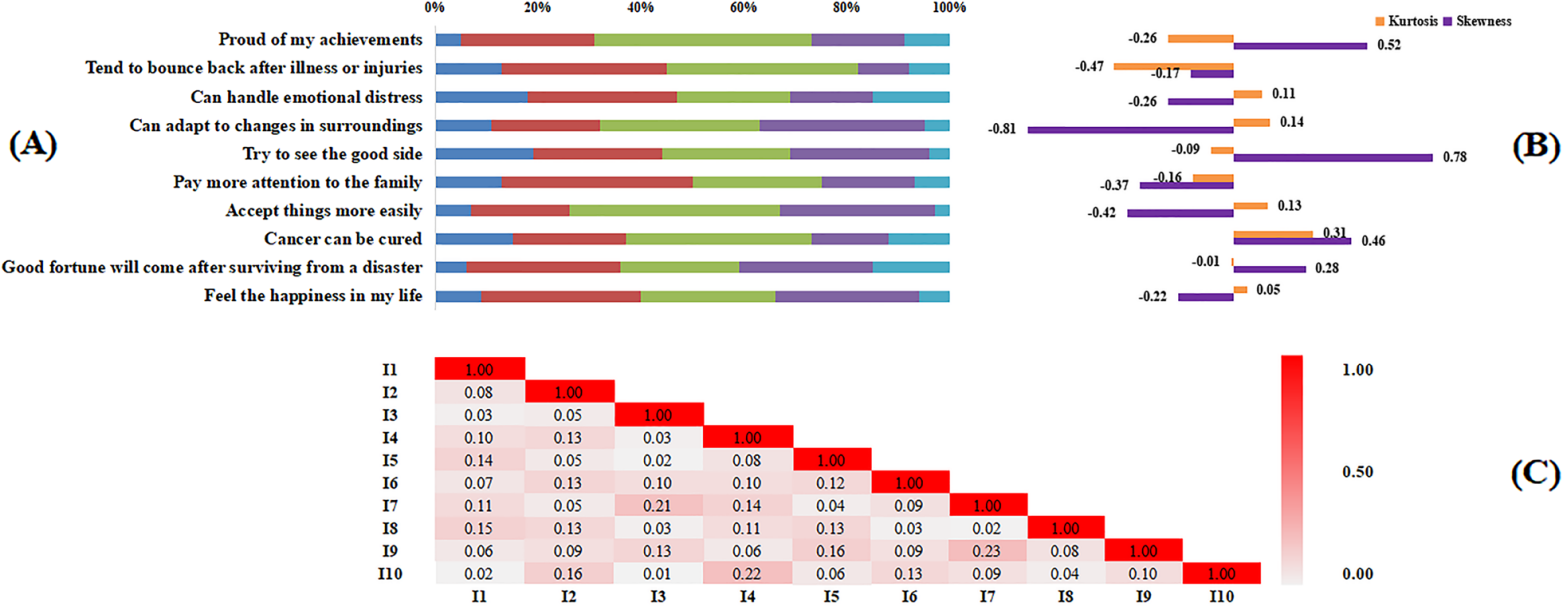
Item distribution and local independence. *C A deeper color indicates a stronger item-pair residuals association. ** **C** A value lower than 0.20 indicated a low risk of systematic fitting problems

The item distribution as well as skewness and kurtosis were visualized in Fig. 1a, b. In addition, item-pair local independence was summarized in Fig. 1c and most associations were lower than 0.20, indicating the local independence hypothesis was satisfied.

### Confirmatory factor analysis-based versus bifactor-based MIRT models

**Fig. 2:**
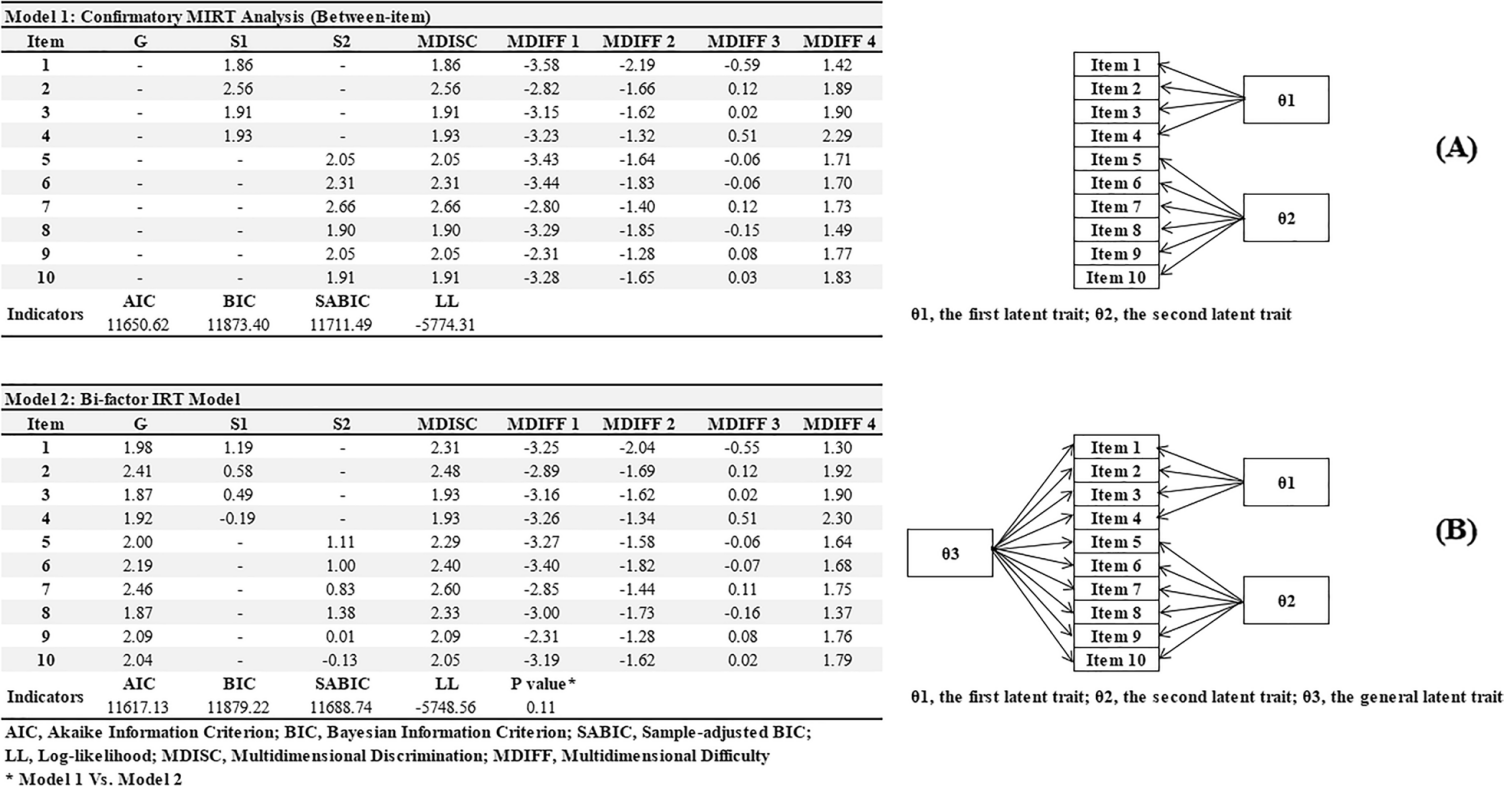
Confirmatory factor analysis-based versus bifactor-based MIRT models

**Fig. 3:**
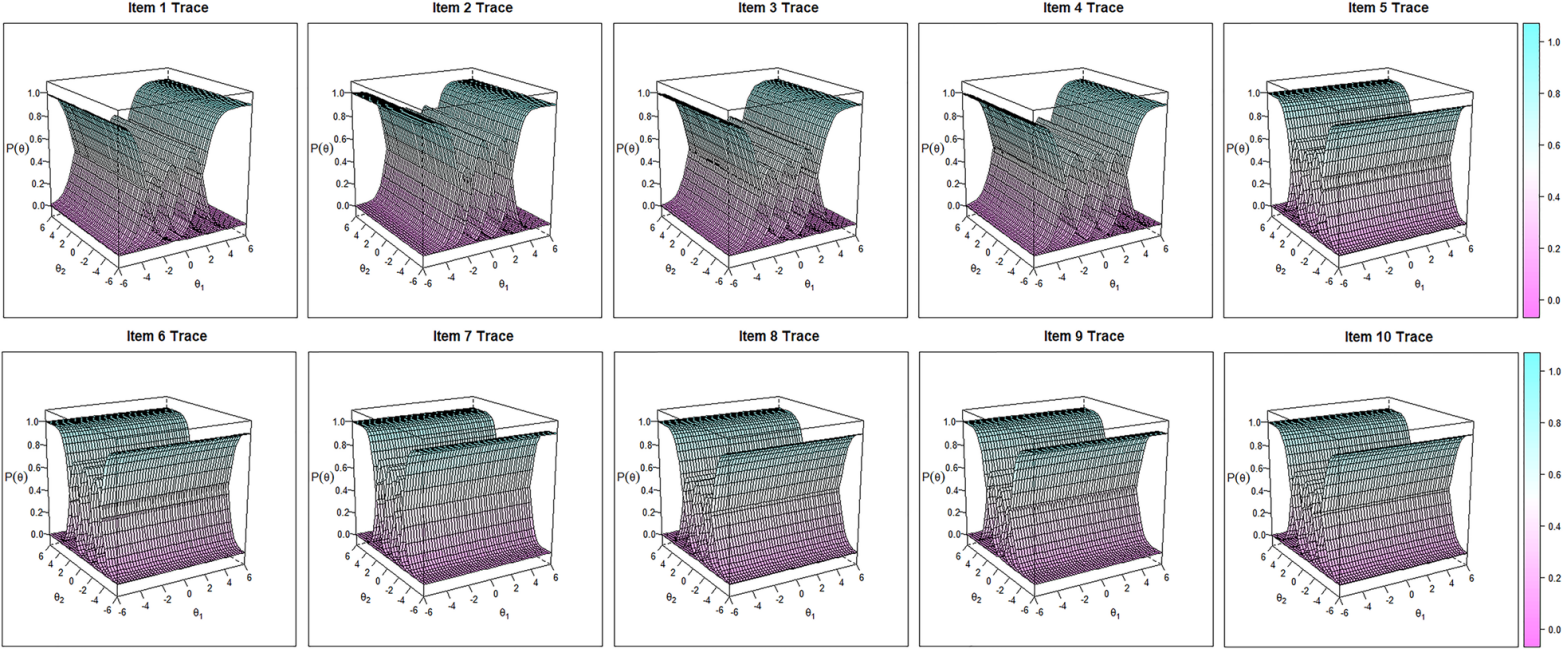
Item trace for RS-SC-10

**Fig. 4:**
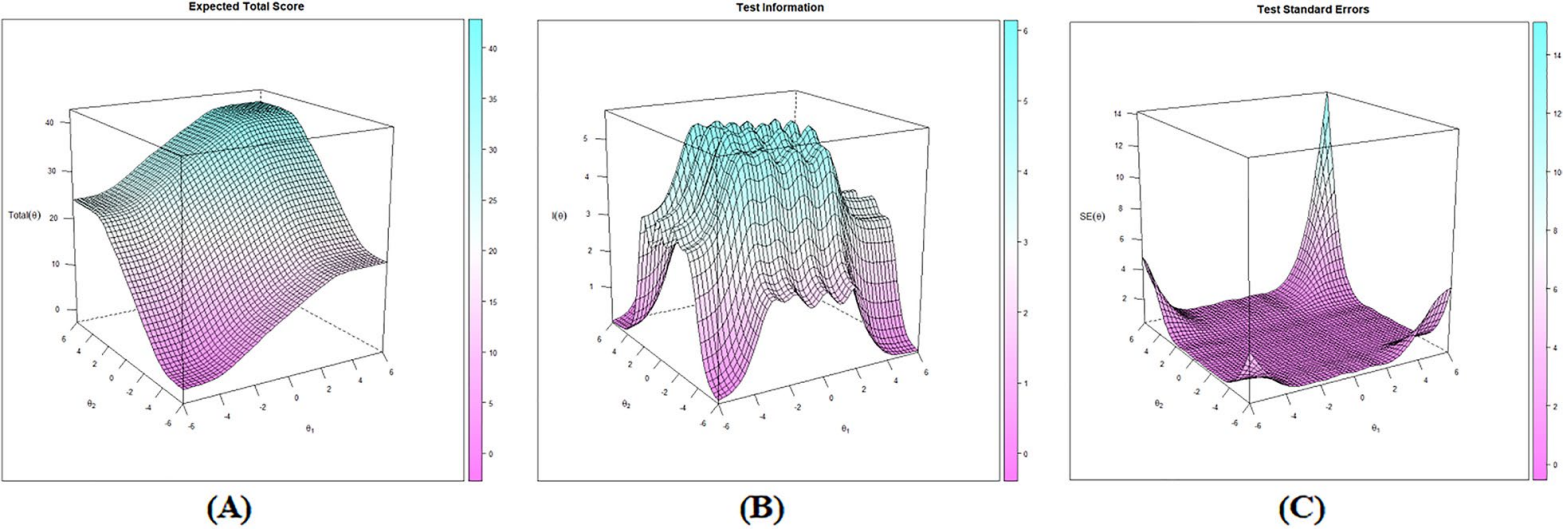
Expected total score, test information and test standard errors for RS-SC-10

Based on the two-factor structure, Confirmatory Factor Analysis-based MIRT model (Model 1, Fig. 2a) and Bifactor-based MIRT model (Model 2, Fig. 2b) had similar fitting indices (AIC, 11,650.62 vs. 11,617.13; BIC, 11,873.40 vs. 11,879.22; SABIC, 11,711.49 vs. 11,688.74) and showed no significant difference (P value = 0.11). However, negative Slope (S1 and S2) values were identified in Model 2 (i.e., − 0.19 for S1 in Item 4, − 0.13 for S2 in Item 10, etc.), indicating the phenomenon of information overextraction. Therefore, according to parsimonious model guideline and fitting indices, we finally chose Model 1 as the optimal MIRT model. All items had MDISC > 1.5 indicating strong multidimensional discriminative abilities against caregivers with different resilience levels. In addition, no disordered threshold was identified in MDIFF (a descending trend as categories increased) and the 5-Likert setting was adequate for RS-SC-10. At last, 10 item traces were plotted to check whether curves were distributed monotonously and orderly along with the theta value and were visualized in Fig. 3. Additional test-related details about Expected Total Score, Test Information and Test Standard Errors were summarized in Fig. 4a–c and RS-SC-10 could provide optimal parameter evaluation in FCs with moderate resilience levels.

### Differential item functions (DIF)

**Fig. 5:**
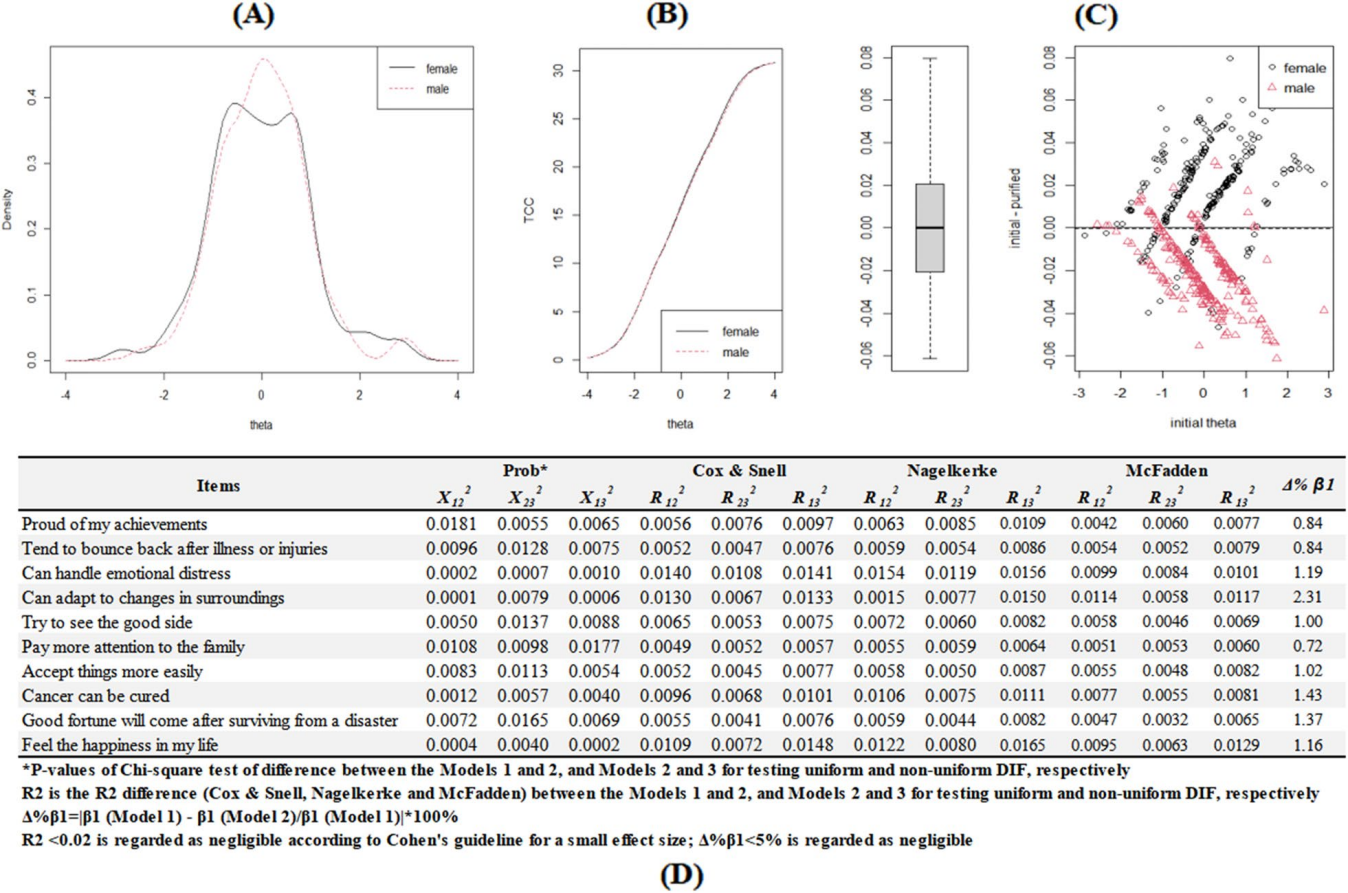
Differential item functioning using iterative hybrid ordinal logistic regression/item response theory and Monte Carlo simulations

In Fig. 5a, the smoothed histograms identified a broad overlap in the distributions, though male caregivers with moderate resilience levels (theta = 0) demonstrated a lower density than female ones. In addition, test characteristic curves (TCCs) were visualized for female and male caregivers in Fig. 5b indicating a minimal difference in the total expected score at the overall test level. At the individual level in Fig. 5c, it indicated that DIF accounted for slightly higher scores (about 0.02 theta) in male caregivers and slightly lower scores (about 0.02 theta) in female caregivers. However, according to empirical threshold values from Monte Carlo simulations in Fig. 5d, both of uniform and non-uniform DIFs were not statistically significant according to different methods and could be ignored. Thus, no DIFs were identified in gender across all items.

### Latent profile analysis and generalized additive model

**Fig. 6:**
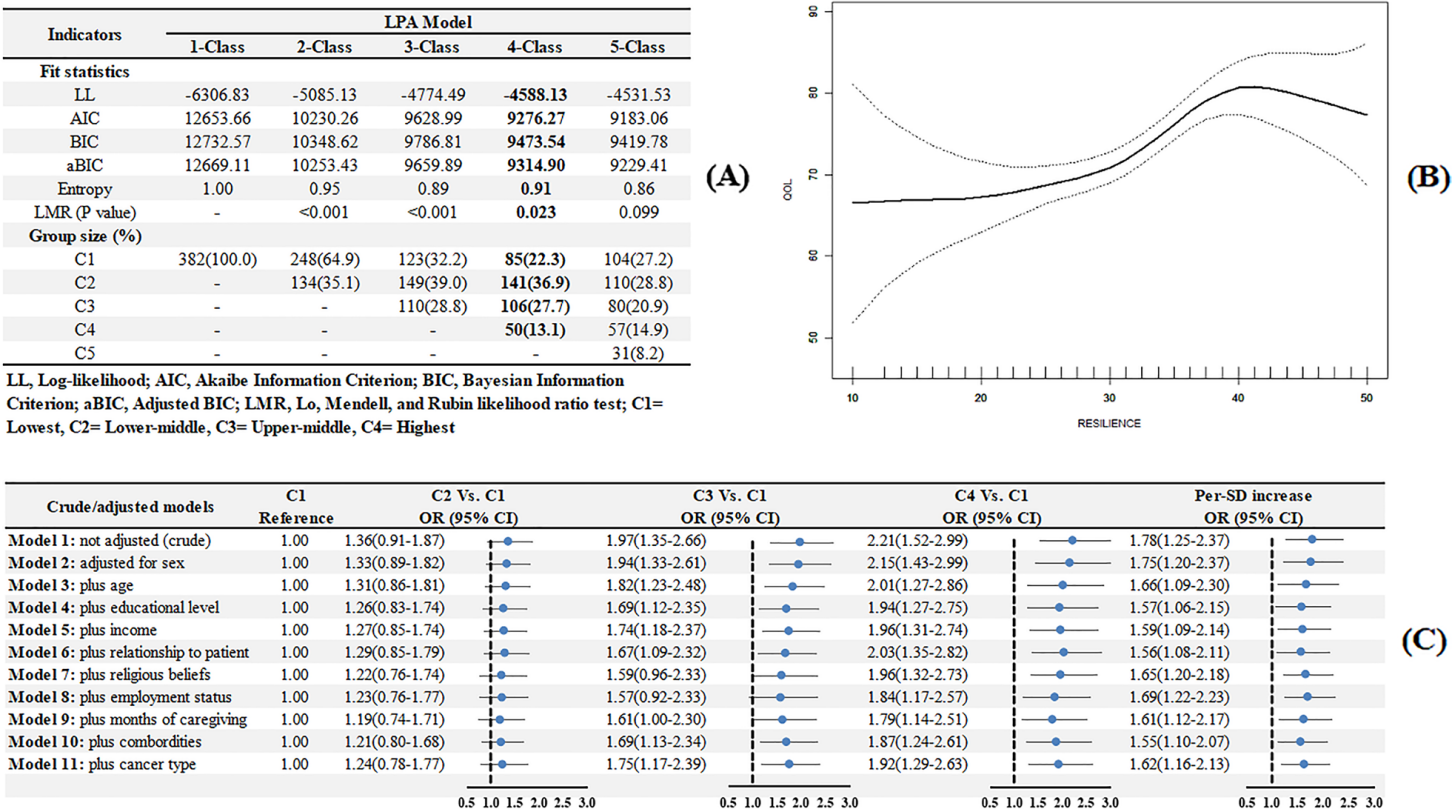
Latent profile analysis and generalized additive model

One to five patterns were fitted by LPA to identify the optimal number of discrete resilience patterns, which were summarized in Fig. 6a. Increasing patterns from one to five provided successive improvements in values of AIC and BIC and the lowest of them were identified at four-pattern model. LMR values for 4-class and 5-class were 0.023 and 0.099 respectively, indicating a 4-class LPA was better than a 5-class one in consideration of parsimoniousness. The entropy value for 4-class model was 0.91, indicating a good classification accuracy (> 95%). Thus, based on the fit statistics and model identifiability, the 4-class solution was retained for further examination, named as C1-C4. As for convergent validity of RS-SC-10, GAM showed that resilience was non-linearly and positively associated with QoL measured by SF-36, which was presented in Fig. 6b. In Fig. 6c, crude, fully adjusted and per-SD OR including 95% CI were summarized in the univariate and multivariate regressions, indicating that the dose–response pattern between resilience and QoL was confirmed (C2 vs. C1, OR = 1.24, 95% CI 0.78–1.77, P = 0.3078; C3 vs. C1, OR = 1.75, 95% CI 1.17–2.39, P = 0.0022; C4 vs. C1, OR = 1.92, 95% CI 1.29–2.63, P = 0.0004). In addition, per-SD increase OR was 1.62, 95% CI 1.16–2.13, P = 0.0019.

## Discussion

In the current study, it validated a new instrument for quantifying resilience of FCs in cancer based on a multidimensional theoretical model. MIRT or full information analysis provides information on item functions by transforming FC’s resilience traits into an interval-level metric, which is more precise than summed scores (ordinal scaling) [15]. The local independence assumption was partly compromised owing to several high (> 0.2) item-pair residuals associations (i.e., items 3 and 7, item 4 and 10. etc.), resulting in a potential biased parameter calculation. However, the problematic item-pair proportions were small (6.7%) and the effect could be ignored. According to cross-loadings between *Generic* and *Shift-Persist* domains in our previous study, two MIRT models were explored including Confirmatory Factor Analysis-based MIRT model and Bifactor-based MIRT model [12]. Finally, a Confirmatory Factor Analysis-based MIRT model confirmed the original two-factor structure (*Generic* and *Shift-Persist*) of RS-SC-10 while the Bifactor-based MIRT model was rejected due to information overextraction. Therefore, a between-item multidimensional theory framework (one item can only measures one latent trait) is more suitable than a within-item one (one item can measure more than two latent traits) in FCs.

As for item functions, the underlying pattern of item responses showed that all 10 items had excellent MDISC (> 1.5) indicating they can well discriminate against FCs with different resilience levels. As such, monotonous thresholds were identified in MDIFF indicating that a 5-Likert option setting was suitable for RS-SC-10. Thus, no category modification or combination should be further adapted. In addition, based on test information, test standard errors and internal consistency, we could conclude that RS-SC-10 was suitable for pattern traits evaluation for FCs with moderate resilience levels, which meant it could be used to distinguish effectively FCs with lower-middle or upper-middle resilience from the entire population.

As for convergent validity of RS-SC-10, it was positively associated with QoL measured by SF-36, which was consistent with previous research [25, 26]. To our interests, resilience was not linearly associated with QoL and four latent resilience subgroups were identified by LPA, resulting in a non-linear dose–response pattern between resilience and QoL (per-SD increase OR = 1.62, 95% CI 1.16–2.13, p = 0.0019).

### Implications for research and clinical practice

According to these findings, resilience-based intervention can be developed to indirectly promote FCs’ QoL especially for FCs with low or moderate resilience levels. However, the clinimetric properties of assessment instruments should be further estimated for RS-SC-10. For example, the Minimum Clinical Important Difference for RS-SC-10 among FCs should be further determined to facilitate RCT-based intervention [27, 28]. In addition, RS-SC-10 may have potential application in adolescents with cancer as well as their caregivers and more research should focus on this vulnerable group [29–32]. For example, actor-partner interdependence model (APIM) can be performed to test the associations between adolescents’ and parents’ resilience, emotional distress, quality of life, etc. [33–35]. Of course, these observational studies can be followed by resilience-based interventions such as our previous BRBC program designed for patients with breast cancer and their caregivers [36, 37]. At last, RS-SC-10 caused less scale burden on FCs and took 63% less time compared with RS-SC-25. Thus, both of patients and FCs could be administered with resilience screening simultaneously in clinical practice especially in outpatients and communities. However, compared to RS-SC-25, RS-SC-10 also has some potential disadvantages. For example, RS-SC-10 can not provide full item information derived from 5-factor structure of RS-SC-25, the validation of RS-SC-25 in future research is warranted.

### Limitations

Several limitations should be considered in the current study. First, there exists the debate about the recommended sample size for MIRT analysis and a sample size more than 500 is recommended to ensure precise parameter estimation [38]. Thus, the statistical power may be compromised in the present study and these findings should be validated in another study with a robust sample size. Second, the item functions are estimated based on the compensatory logistic multidimensional grade response model (MGRM-C), which means *Generic* and *Shift-Persist* are mutually correlated (a higher ability can compensate a lower ability resulting in linear accumulation) [39]. Thus, these findings can not be generalized to tests based on a non-compensatory MGRM (the composite probability is the product of all trait probabilities instead of linear accumulation). More research about non-compensatory MGRM of RS-SC-10 is warranted. Third, the current sample is mostly composed of FCs with caregiver experience less than 12 months (79%) and the generalization of RS-SC-10 in FCs with long term caring should be estimated in future studies. Fourth, the responsiveness of RS-SC-10 to resilience-based intervention should be further estimated which will facilitate its clinical application [40]. At last, 438 caregivers were approached and 56 were excluded for different reasons. Thus, a potential selection bias should be noted as we do not know whether there exists significant difference in their resilience levels.

## Conclusion

RS-SC-10 is a brief and suitable resilience instrument for FCs in cancer. The resilience screening of patients and FCs can be performed simultaneously in clinical practice.

## Supplementary Information

**Additional file 1.** 25-Item Resilience Scale Specific to Cancer.

**Additional file 2.** 10-Item Resilience Scale Specific to Cancer.

## Data Availability

The data that support the findings of this study are available on request from the corresponding author. The data are not publicly available due to privacy or ethical restrictions.

## Abbreviations

RS-SC-10: 10-Item Resilience Scale Specific to Cancer
RS-SC-25: 25-Item Resilience Scale Specific to Cancer
FCs: Family caregivers
MIRT: Multidimensional item response theory
BRCP: Be resilient to cancer program
SF-36: 36-Item Short Form Health Survey
GAM: Generalized additive model
LPA: Latent profile analysis
QoL: Quality of life
BRBC: Be resilient to breast cancer
MGRM-C: Compensatory logistic multidimensional grade response model
MCMC: Markov chain Monte Carlo
LL: Log-likelihood
AIC: Akaike information criterion
BIC: Bayesian information criterion
SABIC: Sample-adjusted BIC
MDISC: Multidimensional discrimination
MDIFF: Multidimensional difficulty
