# Supplementary material for: New resilience instrument for family caregivers in cancer: a multidimensional item response theory analysis
Source: Health Qual Life Outcomes. 2021 Nov 18;19:258. doi: 10.1186/s12955-021-01893-8 (PMC8600888; doi:10.1186/s12955-021-01893-8)
Supplement: Supplementary file 1 — Additional file 1. 25-Item Resilience Scale Specific to Cancer. [file 12955_2021_1893_MOESM1_ESM.docx]

**10-item Resilience Scale Specific to Cancer (RS-SC-10)**

**Instructions:** Please read the items below and indicate how often you agree with these statements over the last 4 weeks. If some specific situations did not occur, imagine about how you would feel if it had happened. There are no right or wrong answers and please circle on the number which most closely described your feelings.

| **Item** | **Content** | **Never** | **Seldom** | **Sometimes** | **Often** | **Always** |
| --- | --- | --- | --- | --- | --- | --- |
| 1 | Proud of my achievements | 1 | 2 | 3 | 4 | 5 |
| 2 | Tend to bounce back after illness or injuries | 1 | 2 | 3 | 4 | 5 |
| 3 | Can handle emotional distress | 1 | 2 | 3 | 4 | 5 |
| 4 | Can adapt to changes in my surroundings | 1 | 2 | 3 | 4 | 5 |
|  | **When you are faced by cancer,** |  |  |  |  |  |
| 5 | Try to see the good side | 1 | 2 | 3 | 4 | 5 |
| 6 | Pay more attention to family | 1 | 2 | 3 | 4 | 5 |
| 7 | Accept things more easily | 1 | 2 | 3 | 4 | 5 |
| 8 | Cancer can be cured | 1 | 2 | 3 | 4 | 5 |
| 9 | I believe that good fortune will come after surviving a disaster | 1 | 2 | 3 | 4 | 5 |
| 10 | Feel the happiness in my life | 1 | 2 | 3 | 4 | 5 |
